# Experimental study on the dynamic properties of low-cement sludge solidified soil under long-term cyclic loading

**DOI:** 10.1038/s41598-025-09987-9

**Published:** 2025-07-10

**Authors:** Jiantao Lin, Wenjun Wang, Xiao Cheng, Haoqiang Pan

**Affiliations:** 1 School of Civil Engineering and Architecture, Zhejiang Sci-Tech University, No. 928 Baiyang Road, Hangzhou, 310018 China; 2 School of Civil Engineering and Architecture, NingboTech University, Qianhu South Road, Ningbo, 315100 China; 3 College of Civil Engineering and Architecture, Zhejiang University, No. 866 Yuhangtang Road, Hangzhou, 310058 China

**Keywords:** Sludge solidified soil, Long-term cyclic loading, Dynamic strength, Accumulative strain, Initial elastic modulus, Civil engineering, Polymers, Environmental impact

## Abstract

The stabilization of waste sludge generated from various engineering projects to produce sludge solidified soil for use as subgrade fill is one of the effective approaches for the resource utilization of construction spoil. However, such solidified soils are typically characterized by relatively low stabilizer content and are subjected to long-term cyclic dynamic loading during their service life. In this study, typical sludge from the Ningbo region was used to prepare cement-stabilized soils with low cement content (4–7%). A series of dynamic triaxial tests under long-term cyclic loading were conducted to systematically investigate the evolution of dynamic strength, cumulative deformation, and initial dynamic elastic modulus. The test results revealed the following: (1) The cumulative deformation of the solidified soil exhibited either a “stable” or a “failure” pattern, with the transition governed by the critical dynamic load, which was strongly influenced by the cement content. (2) Increasing the cement content significantly enhanced the fatigue resistance of the solidified soil; when the content was increased from 4 to 6%, the fatigue life improved by up to 575%. (3) When the cement content exceeded 6%, the growth rates of both fatigue life and initial dynamic elastic modulus slowed markedly, indicating a clear phenomenon of diminishing marginal returns. This study identified 6–7% as the optimal economic range of cement content, achieving an optimal balance between dynamic stability and material cost. These findings provide critical design parameters and performance optimization references for the application of stabilized sludge in road engineering.

## Introduction

Against the backdrop of a low-carbon and green circular economy, the resource reutilization of waste sludge generated from construction activities in soft soil regions can effectively reduce project costs and mitigate environmental impacts. Due to its high water content, low strength, high compressibility, and low permeability, sludge is typically unsuitable for direct use as a construction material and often requires prior treatment^1^. Among various treatment methods, cement stabilization is widely adopted and has proven to be effective. In recent years, the use of stabilized sludge as subgrade fill has emerged as a promising application. Compared with conventionally stabilized in-situ foundations, solidified sludge used as subgrade fill exhibits two notable characteristics: (1) a generally low stabilizer (cement) content, and (2) the need to withstand long-term cyclic traffic loading during its service life.

The static properties of solidified soil are significantly influenced by the stabilizer content. Horpibulsuk et al.^2–6^ conducted experimental studies and found that the strength of cement soil exhibited three characteristic zones as the cement content increased: the inactive zone (Zone-I), the active zone (Zone-II), and the inert zone (Zone-III). Zone-II is the range of cement content typically used in conventional soft foundation treatment projects, and its strength growth rate is significantly higher than that of Zone-I. When the cement content is in the lower Zone-I, the mechanical behavior of the solidified soil differs significantly from that of the conventional cement content in Zone-II. When the stabilizer content is low, the solidified soil exhibits characteristics that are different from those of the conventional cement content. Soheila et al.^7^ found that the cement content significantly affects the consolidation coefficients (Cc and Cs) of soil. Compared to a low cement content (5%), a conventional cement content (15%) resulted in a reduction of 84% and 89% in compression index and expansion index, respectively, after 28 days of curing. Zhang et al.^4,5^ indicated that when the stabilizer content is low, the strength of solidified soil increases non-linearly with the stabilizer content. The boundary cement content (awc) between Zones I and II is related to soil type, moisture content, and the type of stabilizer. Gyeongo Kang et al.^8^ studied the strength development of Tokuyama Port sludge solidified soil in the early stages with cement contents of 2%, 4%, 6%, 10%, and 15%. They found that when the cement content was low, the strength increased very slowly. However, when the cement content reached the conventional range, the strength increased significantly, especially in the early curing stage. Sun et al.^9^ pointed out that since the strength requirements for subgrade fill are not very high, the stabilizer content is often low. Different stabilizer contents result in variations in the structural strength and mechanical properties of the solidified soil. Tong et al.^10^ conducted damage tests on cement soil and concluded that, unlike Zone-I, the strength of solidified soil in Zone-II increased rapidly. The hydration products of cement serve as the skeletal structure, and the hardened body exhibits linear elastic behavior. Chen et al.^11^ found that as the cement content increased, the water content of cement soil in Zone-I gradually decreased, the plastic limit increased, and the liquid limit and plasticity index initially increased and then decreased. Zhang et al.^12^ concluded that when the cement content was low, the expansion index slightly decreased, and as the cement content increased, the expansion index decreased significantly. Khajeh et al.^13–18^ found that natural volcanic ash materials, such as zeolite, can significantly improve unconfined compressive strength (UCS), enhance plasticity, and reduce expansion when used as partial replacements for cement. The addition of lightweight materials like EPS beads also helps reduce the weight while maintaining the mechanical performance of the subgrade fill, further expanding the potential applications of low-cement solidified soil. Sasanian et al.^19^ summarized that different cement contents significantly affect the undrained shear strength and sensitivity (St). At low cement contents, the effect of cement on strength increase and sensitivity variation is limited, while at high cement contents, there is a more substantial increase in both strength and sensitivity. The above studies show that low-cement solidified soils exhibit significantly different characteristics from conventional cement solidified soils in terms of performance. The boundary cement content (*a*_wc_) is influenced by various factors such as soil type, moisture content, and the type of stabilizer.

While attention has long been given to the static properties of solidified soils, a considerable number of experimental studies have also been conducted on their dynamic behavior under conventional binder contents. These studies primarily focus on how curing duration, type of binder, and loading frequency influence the dynamic shear modulus, dynamic elastic modulus, and damping ratio of specimens^20–24^. Regarding stabilization materials, various modifiers have been shown to significantly affect the dynamic response. Di et al.^25^ found that incorporating superabsorbent polymer (SAP) effectively improved the failure mode and reduced the cumulative deformation of cement-stabilized soils with low binder content. Du et al.^26^ confirmed that increasing the cement content enhances the dynamic elastic modulus (*E*_max_) and reduces the damping ratio, thus improving resistance to dynamic loading. Zhang et al.^27^ investigated the dynamic characteristics of sludge stabilized with lignin. The experiments revealed that substituting lignin within 10% did not significantly compromise the dynamic stiffness of the soil under long-term curing. Furthermore, lignin offers environmental and cost advantages. The higher the replacement ratio, the greater the damping ratio observed. Fahoum et al.^28^ demonstrated that lime stabilization effectively enhances the dynamic performance of cohesive soils, with an optimal binder content identified. In terms of fatigue performance, Biswal et al.^29^ conducted fatigue tests on cement-stabilized granular red soil under conventional binder content. The results showed that the elastic modulus of the specimens decreased progressively with the number of loading cycles and failed completely when the stress reached 25% of the maximum. A linear relationship was observed between fatigue life and stress ratio. Zhang et al.^30^ explored the key influencing factors and underlying mechanism of fatigue failure in cement-stabilized soils. Using ultrasonic testing, they examined the fatigue damage process and found significant variability in fatigue life. Moreover, the fatigue life tended to increase with higher stress levels and longer curing periods. Xin et al.^31^ conducted dynamic triaxial tests on specimens with cement contents ranging from 0 to 10%. The study showed that lower cement contents resulted in larger hysteresis loop areas and more pronounced cumulative plastic deformation under cyclic loading. The trends in dynamic elastic modulus and cumulative plastic strain confirmed the performance degradation under long-term low-frequency cyclic loading. A synthesis of current literature reveals two major limitations in the study of the dynamic properties of solidified soils: (1) the number of loading cycles applied in most experiments remains limited (typically only a few thousand), insufficient to reflect long-term cyclic loading effects; and (2) research has predominantly focused on solidified soils with conventional binder contents, with inadequate attention given to the dynamic behavior of low-binder solidified soils.

In summary, addressing the current research gap concerning the dynamic behavior of low-cement-content stabilized sludge under long-term cyclic loading, this study investigates typical dredged sludge from the Ningbo region. A series of dynamic triaxial tests was conducted to systematically examine the dynamic characteristics of cement-stabilized soil with low binder content subjected to prolonged cyclic loading. Particular emphasis was placed on the development of dynamic strength, the evolution of cumulative plastic strain, and the variation trend of the initial dynamic elastic modulus. Based on both the dynamic performance indices and economic evaluation of the stabilized sludge, the optimal cement content suitable for subgrade applications was proposed. The findings of this study provide essential theoretical guidance and data support for the design and practical application of low-binder-content stabilized sludge in subgrade engineering. This contributes to the efficient reutilization and sustainable management of dredged waste sludge.

## Experimental design and methodology

### Experimental materials

The soil used in this experiment was collected from a construction site located in Meixu Subdistrict, High-tech Zone, Ningbo. The tested material was a typical thick, shallow-layer soft soil commonly found in the Ningbo area, identified as silty clay with high silt content. According to the Unified Soil Classification System (USCS), the soil was classified as CL, corresponding to low-plasticity clay. The soil was gray in color, exhibited a flow plastic state, had a thick-layered structure, and demonstrated high compressibility. Additional physical properties are summarized in Table 1. The stabilizer used in this study was Conch-brand P.O 42.5 ordinary Portland cement.

**Table 1:** Basic physical properties of clay.

Natural Density /g·cm^3^	Natural Moisture Content /%	Dry Density /g·cm^− 3^	Liquid Limit/%	Specific gravity (Gs)	Plastic Limit/%	Porosity Ratio	Plasticity Index
1.73	48.0	1.17	38.2	2.73	21.7	1.266	16.5

### Experimental plan

#### Unconfined compressive strength test

Cubic specimens with dimensions of 70.7 mm × 70.7 mm × 70.7 mm were first prepared using various cement contents. Unconfined compressive strength (UCS) tests were conducted using a STYE-300E fully automatic cement flexural-compressive testing machine. The loading rate was set at 0.3 mm/min. Based on the UCS test results, the threshold cement content, denoted as *a*_wc_, was identified to define the low-content range. The cement content *aw* was calculated according to Eq. (1).

1 $${a_{\text{w}}}=\frac{{{\text{Cement Mass}}}}{{{\text{Wet Soil Mass}}}} \times {\text{100\% }}$$

The specific testing protocol is outlined in Table 2.

**Table 2:** Unconfined compressive strength test plan.

Cement Content(a_w_)/%	Water-to-Cement Ratio w/c	Curing Age/d	Number of parallel specimens
2、3、4、5、6、7、8、9、10、11、12	0.5	28	3

#### Dynamic triaxial test

Dynamic triaxial tests were carried out on cement-stabilized sludge within the low cement content range. The tests were conducted using a servo-motor-controlled bidirectional dynamic triaxial apparatus manufactured by GDS Instruments (UK), with a maximum axial load capacity of 10 kN, a maximum confining pressure of 2 MPa, and a frequency capacity of up to 2 Hz. Standard cylindrical specimens with a diameter of 50 mm and a height of 100 mm were used. During the tests, undrained shear conditions were applied, with a consolidation stress ratio (*K*_c_) of 1. A sinusoidal waveform was adopted for cyclic loading. Previous studies have shown that within the frequency range of 1 to 5 Hz, loading frequency has negligible influence on the dynamic stress–strain behavior and dynamic elastic modulus of stabilized soils^32–34^ Moreover, considering the dynamic loading characteristics typically experienced by subgrade structures, and the widespread use of 1 Hz as a representative loading frequency in laboratory studies of subgrade materials, a loading frequency of 1 Hz was consistently adopted in this study.

Prior to formal loading, all specimens underwent a standardized preparation procedure: Back-pressure saturation was performed until the B-value exceeded 0.95. Subsequently, isotropic consolidation was carried out under the specified confining pressure, with completion determined by the stabilization of volumetric strain rate. After consolidation, the specimens were subjected to the predefined sinusoidal cyclic loading under undrained conditions. The entire testing process—including saturation, consolidation, shearing, and data acquisition—was automatically controlled by GDSLAB software, ensuring high precision in operation and reliability in data collection.

To simulate the stress conditions experienced by stabilized soils at different depths within subgrade engineering applications, three levels of confining pressure (*σ*_3_) were applied in this study: 50 kPa, 100 kPa, and 150 kPa. This range of confining pressures, selected based on relevant literature^35,36^, represents the lateral stress conditions induced by the self-weight of the overlying soil and traffic-induced loads in subgrade structures, and also covers potential overload scenarios. Under each confining pressure condition, cyclic deviatoric stress amplitudes (*σ*_d_) of 200 kPa, 250 kPa, 300 kPa, and 400 kPa were applied. According to field measurements and related studies^35–37^, this range of dynamic stress amplitudes represents typical vertical dynamic loading conditions that may be encountered at the surface and shallow layers of highway subgrades under heavy traffic. All specimens were tested after 28 days of curing under standard laboratory conditions. In accordance with the Standard for Soil Dynamics Testing Methods (GB/T 50123 − 2019), a strain-based failure criterion was adopted, whereby a specimen was considered to have failed when the axial strain (*ε*_p_) reached 5%.

### Sample preparation and curing

The soil samples were air-dried, crushed, and passed through a 2 mm sieve for subsequent use. During specimen preparation, wet soil was prepared based on the initial moisture content of the sludge, which was 48.0%. For each specimen, the required mass of sieved dry soil (*m*₁) was accurately weighed. The total amount of water required for each specimen (*w*₁) was calculated based on the initial moisture content using the following formula:

2 $${w_1}={m_1} \times 48\%$$

Meanwhile, based on the predetermined cement dosage *a*_w_ and a water-to-cement ratio of 0.5 (mass of water/mass of cement), the required water amount *w*_2_ for preparing the cement paste is calculated as follows:

3 $${w_{\text{2}}}{\text{=}}\left( {{m_1}+{w_{\text{1}}}} \right) \times {a_w} \times 0.5$$

To ensure that the target moisture content is ultimately achieved and that the cement is uniformly distributed, a stepwise water addition method was employed:

a. Pre-wetting the soil: the amount of water (*w*_3_) to be directly added to the dry soil was calculated as the total water content minus the amount used for preparing the cement slurry:

4 $${w_3}={w_1} - {w_2}$$

Using a spraying device, the calculated amount of distilled water (*w*_3_) was evenly sprayed onto the weighed dry soil and thoroughly mixed. The wetted soil was then sealed in a plastic bag and left to soak for at least 8 h to ensure sufficient moisture penetration and distribution between the soil particles.

b. Preparation and addition of the cement slurry: The calculated amount of P.O 42.5 cement was weighed, and the calculated amount of distilled water (*w*_2_) was added, followed by thorough mixing to form the cement slurry. The prepared cement slurry was then added to the pre-wetted soil.

The wetted soil containing cement slurry was thoroughly mixed using a mechanical mixer until the color and moisture of the mixture were uniform. The mixed solidified soil was then placed in three layers into a standard three-lobed cylindrical mold with an inner diameter of 50 mm and a height of 100 mm. After each layer was added, the surface was scarified to enhance interlayer bonding and reduce interface effects. The mass of each layer was strictly controlled to ensure that the final compacted specimen reached the target dry density. Once the specimen preparation was complete, it was immediately wrapped in plastic film and placed in a standard curing chamber (temperature: 20 ± 2 °C, relative humidity > 95%) for curing, with a curing period of 28 days.

## Determination of the dividing binder content *a*_wc_ for sludge solidified soil

**Fig. 1:**
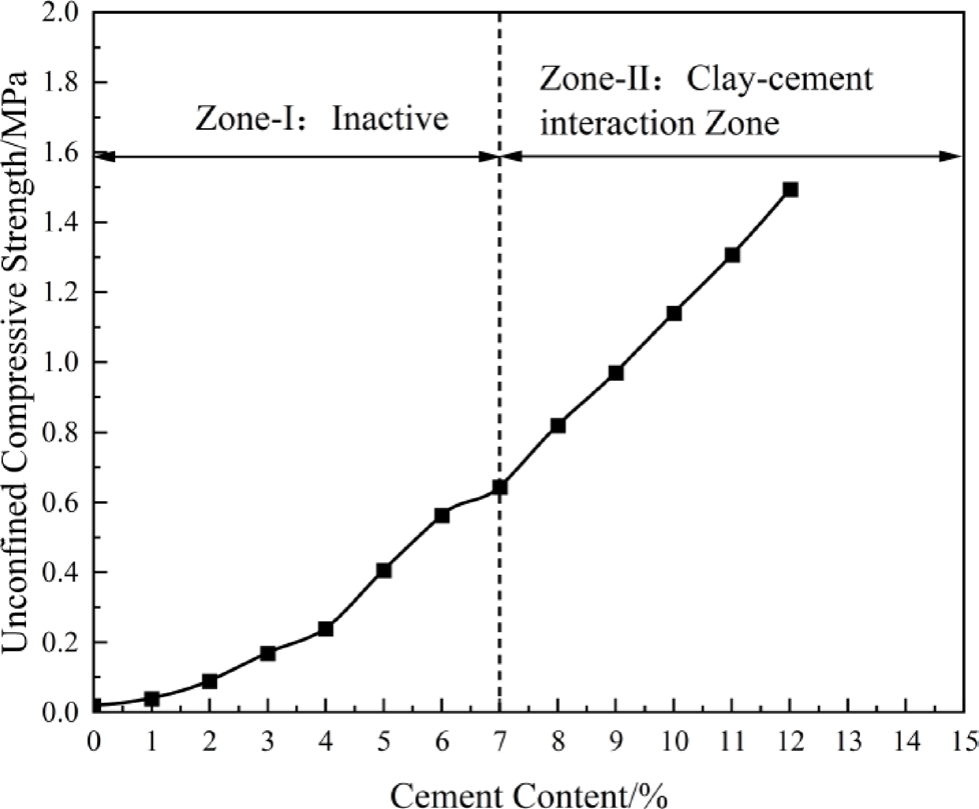
Variation of Unconfined Compressive Strength with Cement Content.

The critical cement content (*a*_wc_) varies with the type of soil, moisture content, and type of cement. To determine the working range of low-cement solidified soil in this study, the value of *a*_wc_ must first be determined based on the unconfined compressive strength (*q*_u_). Figure 1 shows the relationship between the critical cement content (*a*_w_) and unconfined compressive strength (*q*_u_) for the 28-day aged sludge solidified soil obtained from experiments. From Fig. 1, it is clear that the strength development exhibits two distinct phases: when *a*_w_ increases from 0 to 7%, *q*_u_ rises from 0.02 MPa to 0.65 MPa with a small increase and a nonlinear growth pattern, with the growth rate gradually decreasing. However, when *a*_w_ increases from 8 to 12%, the compressive strength increases significantly from 0.82 MPa to 1.49 MPa, showing a linear growth pattern.

**Fig. 2:**
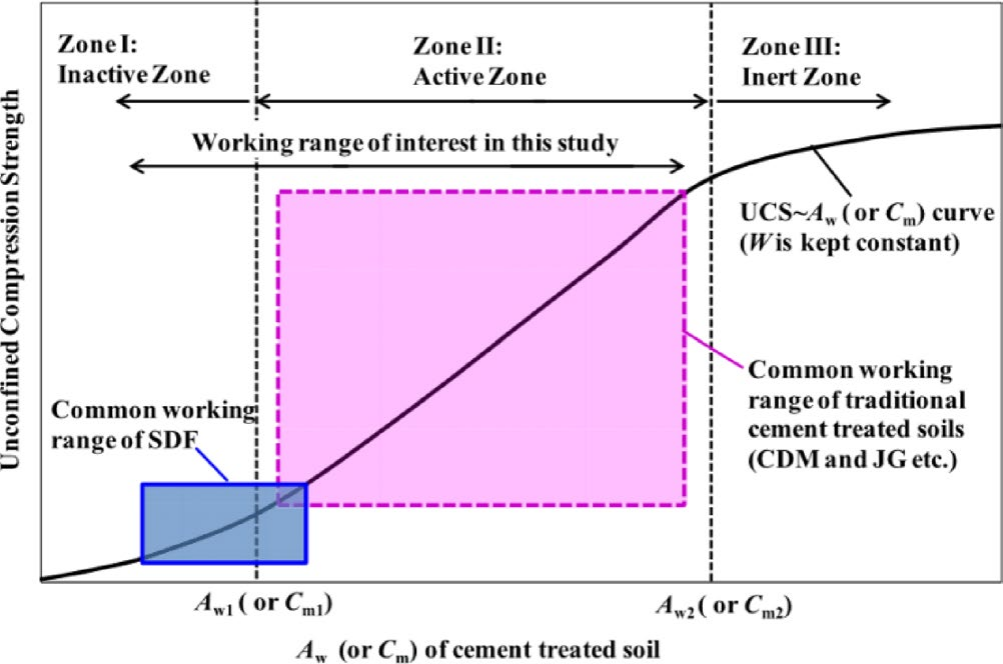
Working Range Zone of Cement Solidified Soil^4^.

Based on the experimental data and referring to the cement-solidified soil working range zoning diagram (Fig. 2), the critical cement content for the 28-day aged sludge solidified soil in this study was determined to be *a*_wc_ =7%. Sun Haichao’s study^9^ determined that *a*_wc_ =8.5%. In Zone I (*a*_w_=3−7%), the average compressive strength growth rate was 8.9%, while in Zone II (*a*_w_ =8−12%), the average compressive strength growth rate was 16.8%. It can be observed that the strength growth rate in the conventional cement content zone is 1.88 times that in the low-cement working zone, indicating a significant contribution from the higher cement content.

From the perspective of the solidification mechanism, when *a*_w_ < *a*_wc_, in the low binder content zone (Zone I), the strength growth rate of the solidified soil is relatively low and increases slowly. This is mainly due to the limited cement hydration products (such as C-S-H gel), which are insufficient to form a continuous and effective cementing network between the widely distributed soil particles. The cementing effect can only serve to locally fill gaps and weakly bond particles, leading to a slow increase in macroscopic strength. However, when *a*_w_ > *a*_wc_, entering Zone II, sufficient hydration products can generate a continuous and more rigid skeleton structure, firmly bonding the originally loose soil particles together, resulting in a significant increase in the macroscopic strength of the solidified soil.

## Analysis of dynamic properties test results

### Dynamic strength

**Fig. 3:**
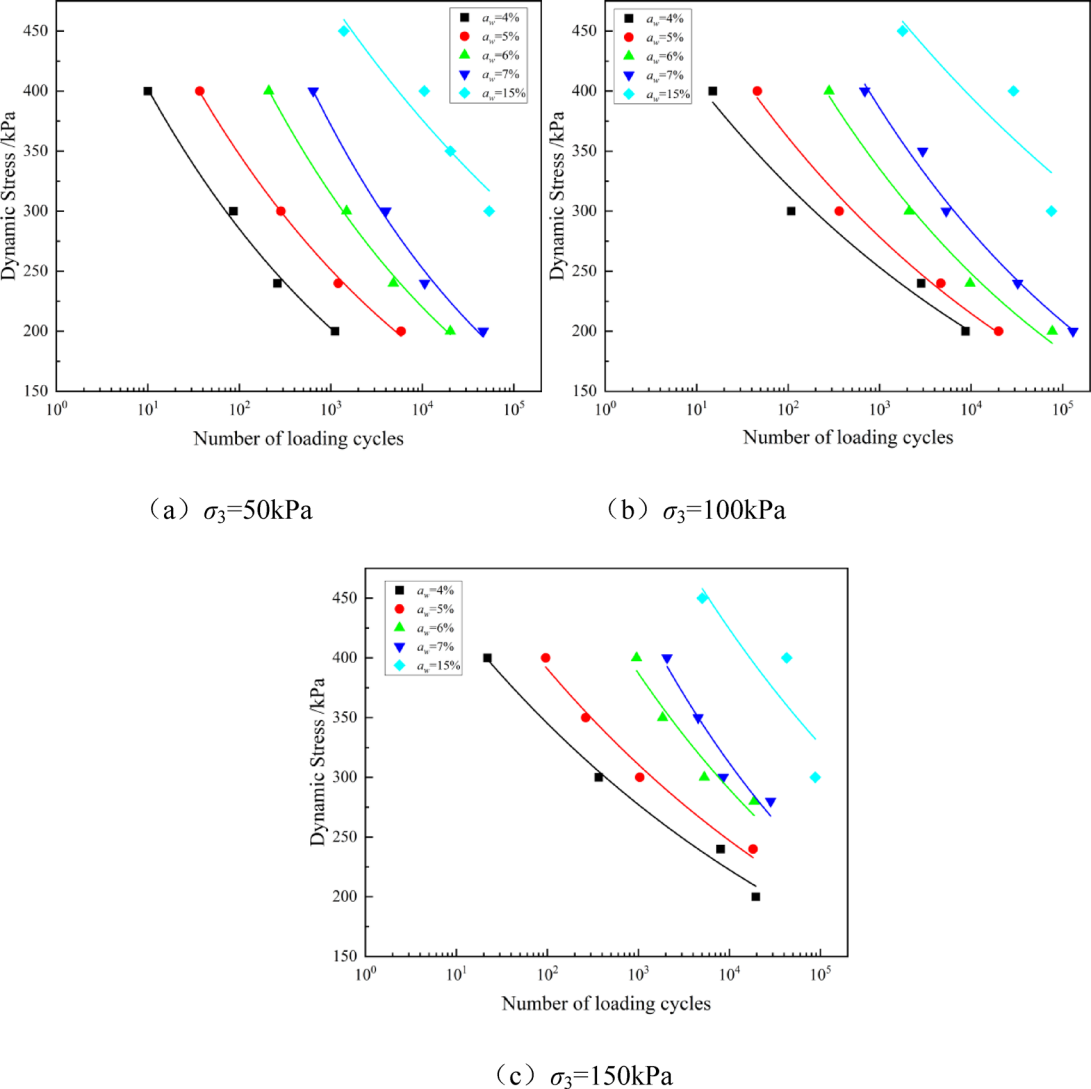
Relationship curve of dynamic strength of solidified soil at different cement contents.

**Fig. 4:**
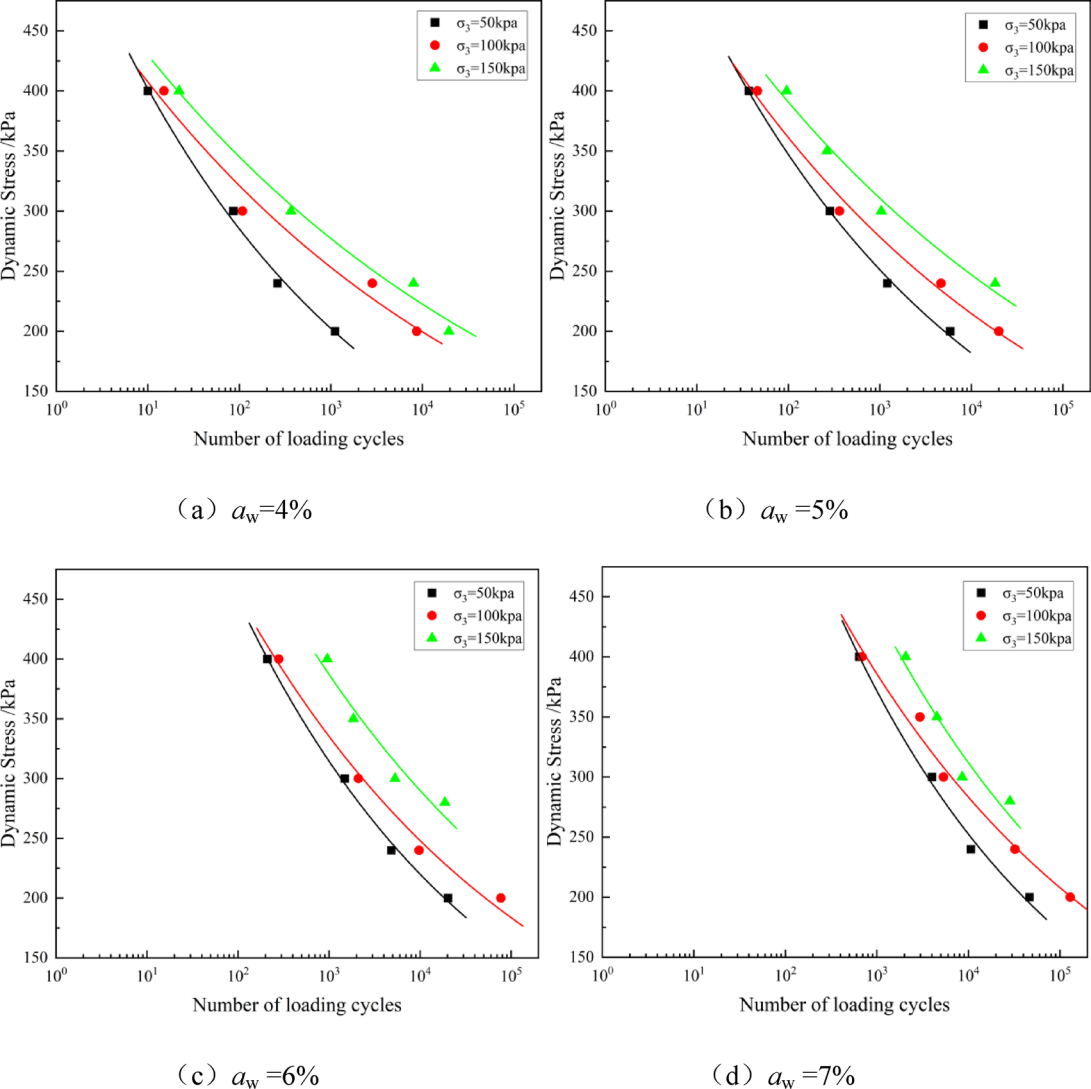
Relationship curve of dynamic strength of solidified soil at different confining pressure.

The dynamic strength of soil is typically defined as the dynamic load amplitude corresponding to the sample’s failure when a specified cyclic load is applied. Dynamic strength is a key indicator for evaluating the engineering performance of solidified soils under cyclic loading, and it holds significant practical importance. Based on the low cement content working range derived from Fig. 1, the dynamic characteristics of solidified soil with *a*_w_ =4−7% were investigated through dynamic triaxial tests, and the results were compared with those of conventional cement content *a*_w_ =15%. Figures 3 and 4 show the dynamic strength (dynamic load *σ*_d_ - failure cycles *N*_f_) relationship curves obtained under different cement contents and confining pressures using the GDS dynamic triaxial test apparatus.

Figure 3 shows the relationship between dynamic load and failure cycles of solidified soil at different cement contents. As shown in the figure, the dynamic strength of solidified soil increases with the increase in cement content. This is mainly attributed to the cement hydration products, such as calcium silicate hydrate (C-S-H) and calcium aluminate hydrate (C-A-H), which form a large amount of gel-like products between the sludge particles. These gel products fill the pores of the soil, bonding the loose sludge particles together into a cohesive whole, thereby constructing a denser and more stable spatial network structure (i.e., the cemented soil cementing network), significantly improving the material’s ability to resist dynamic loading. *N*_*f*_ increases as the dynamic load *σ*_d_ decreases, indicating that the failure of the solidified soil under cyclic loading is an accumulation process. Lower dynamic loads allow the material to withstand more loading cycles.

Taking *σ*_3_ = 500 kPa and *σ*_d_ = 300 kPa as an example, in the low cement content range, when the cement content increases from 4 to 7%, the corresponding failure cycles *N*_*f*_ increased by 231.4%, 423.16%, and 169.01%, respectively. Among these, the most significant increase in *N*_*f*_ occurred when the cement content increased from 5 to 6%, indicating that the effect of cement content on dynamic strength improvement was most evident in this range. However, when the cement content further increased from 7 to 15%, at the same dynamic stress, the total increase in *N*_*f*_ was only 406.88%, with the average growth rate lower than that in the 4-7% range. This suggests that as the cement content continues to increase, its effect on *N*_*f*_ becomes less pronounced, showing a diminishing marginal return. This diminishing marginal effect could be related to several factors: on one hand, as the cement content increases, the binding water or reactive soil components available for effective cementation may become saturated in the soil per unit volume, and excess cement may fail to form effective soil-cement bonds. Some cement particles might only undergo self-hydration to form agglomerates instead of a uniformly distributed gel network. On the other hand, the cementing efficiency no longer significantly improves the macromechanical performance of the overall structure as it does in the low-cement content range. The dynamic strength curve of cemented soil at conventional cement content is significantly higher than at other cement contents, indicating that under the same conditions and *N*_*f*_, the higher the cement content, the greater the ability to withstand dynamic loading. The slope of the fitted curve is relatively small, suggesting that the reduction in dynamic load has a relatively small effect on the number of failure cycles. Solidified soil at high cement content shows more stable dynamic strength performance. When *σ*_3_ = 100 kPa and *σ*_d_ = 200 kPa, the corresponding failure cycles for cement contents of 4%, 5%, 6%, and 7% were 8,683, 20,000, 77,200, and 130,000, respectively. It can be observed that when the cement content exceeds 5%, the failure cycles surpass 10,000, greatly improving the material’s ability to resist fatigue loading. In conclusion, for subgrade engineering subjected to long-term cyclic loading, especially under lower dynamic loading levels, selecting a cement content of 6-7% can significantly enhance the material’s fatigue resistance while ensuring good cost-effectiveness.

As shown in Fig. 4, under the same cement content level, an increase in confining pressure significantly enhances the dynamic strength of the solidified soil. Taking the solidified soil with *a*_w_ = 6% as an example, when *N*_*f*_ = 1000 cycles, the dynamic loads corresponding to confining pressures of 50 kPa, 100 kPa, and 150 kPa are approximately 300 kPa, 350 kPa, and 400 kPa, respectively. The increase in confining pressure allows the solidified soil to achieve the same fatigue life under higher dynamic stresses, or a longer fatigue life under the same dynamic stress. At the same time, as confining pressure increases, the dynamic strength curve flattens, meaning the rate at which Nf increases with σd slows down. This indicates that higher confining pressure significantly enhances the fatigue resistance of the solidified soil, especially under higher stress levels.

A power function was used to fit the dynamic strength curve^38^, The fitting equation is:

5 $${\sigma _d}=A{N_f}^{B}$$

In the equation, *A* and *B* represent the fitting coefficients, with units of 1.

The fitting results are listed in Table 3.

**Table 3:** Fitting parameters of dynamic strength of solidified Soil.

Cement content /%	Confining pressure /kPa	Power function fitting parameters		
		*A*/100	*B*	*R* ^2^
4	50	5.66	-0.15	0.992
	100	5.17	-0.10	0.960
	150	5.35	-0.10	0.982
5	50	6.63	-0.14	0.998
	100	6.08	-0.11	0.985
	150	6.18	-0.10	0.976
6	50	9.18	-0.16	0.995
	100	8.27	-0.13	0.986
	150	9.56	-0.13	0.909
7	50	11.84	-0.17	0.990
	100	9.75	-0.13	0.981
	150	8.94	-0.15	0.902

**Fig. 5:**
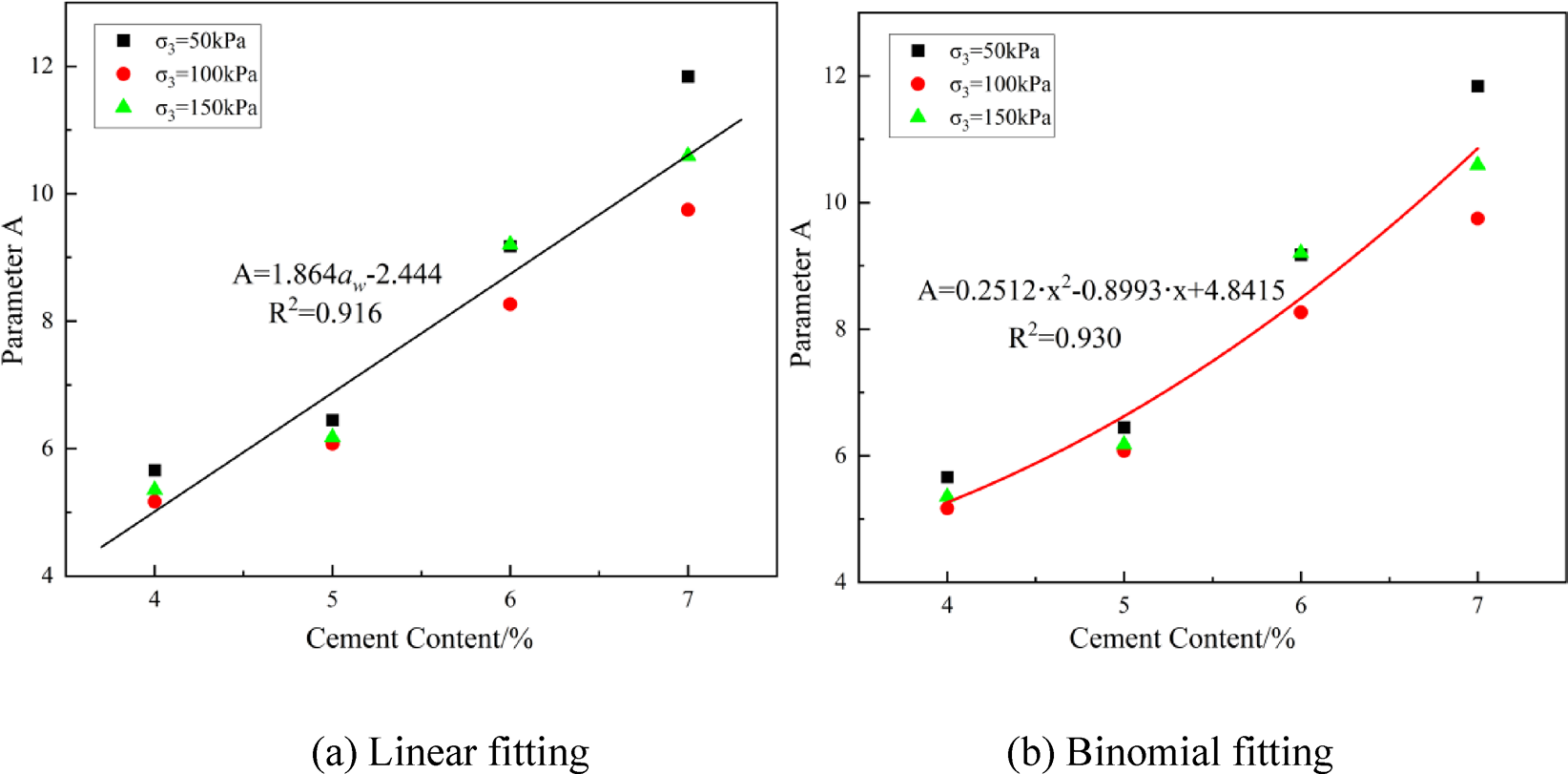
Fitting curves for two different parameter A values.

From the table, it can be observed that the fitting results of the power function for the dynamic strength curve of the solidified soil show good correlation, with *R*² values greater than 0.9. A further analysis of the relationship between fitting parameters *A*, *B* and the dosage aw reveals that the *B* values are distributed within a narrow range of -0.10 to -0.17, so an average *B* value of -0.13 is used. The two different fitting results for parameter *A* are shown in Fig. 5. As shown in Fig. 5, the binomial fitting performs better than the linear fitting, indicating that the parameter *A* of low-cement solidified soil exhibits more significant nonlinear variation characteristics, which is different from the linear relationship observed in conventional solidified soil^22^.

Further, the unified expression for the dynamic strength curve of solidified soil can be obtained as:

6 $${\sigma _{\text{d}}}=\left( {2.444+1.864{a_{\text{w}}}} \right){N_f}^{{ - 0.130}}$$

This formula can be used to predict the dynamic strength curve of sludge solidified soil at different cement contents, and thereby determine the critical failure cycles under various dynamic loads, providing a basis for the preliminary selection of cement content and fatigue design of the subgrade structure.

### Accumulative strain

**Fig. 6:**
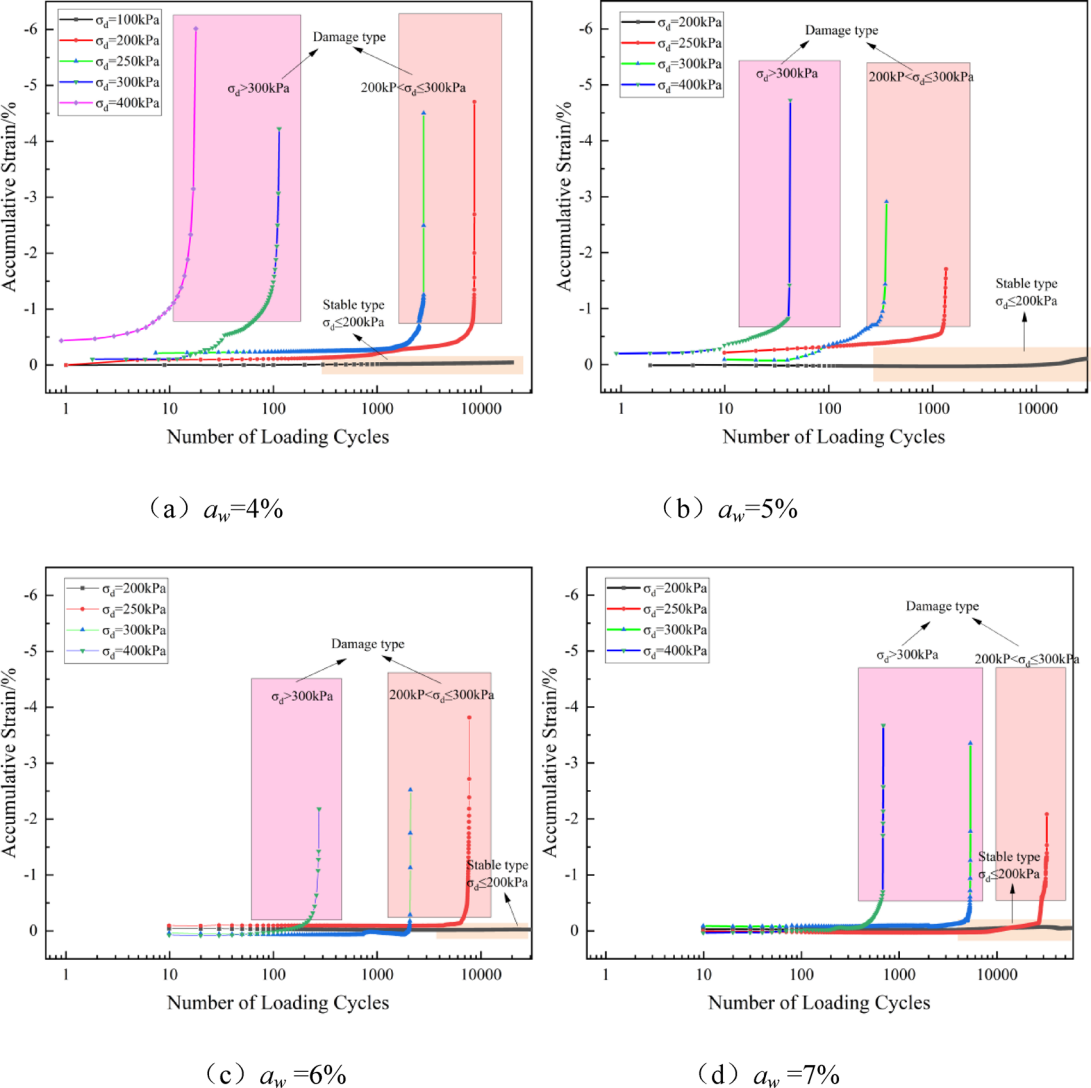
Relationship curve of cumulative strain-number of loading cycles versus dynamic load amplitude at different cement contents (*σ*_3_ = 100 kPa).

**Fig. 7:**
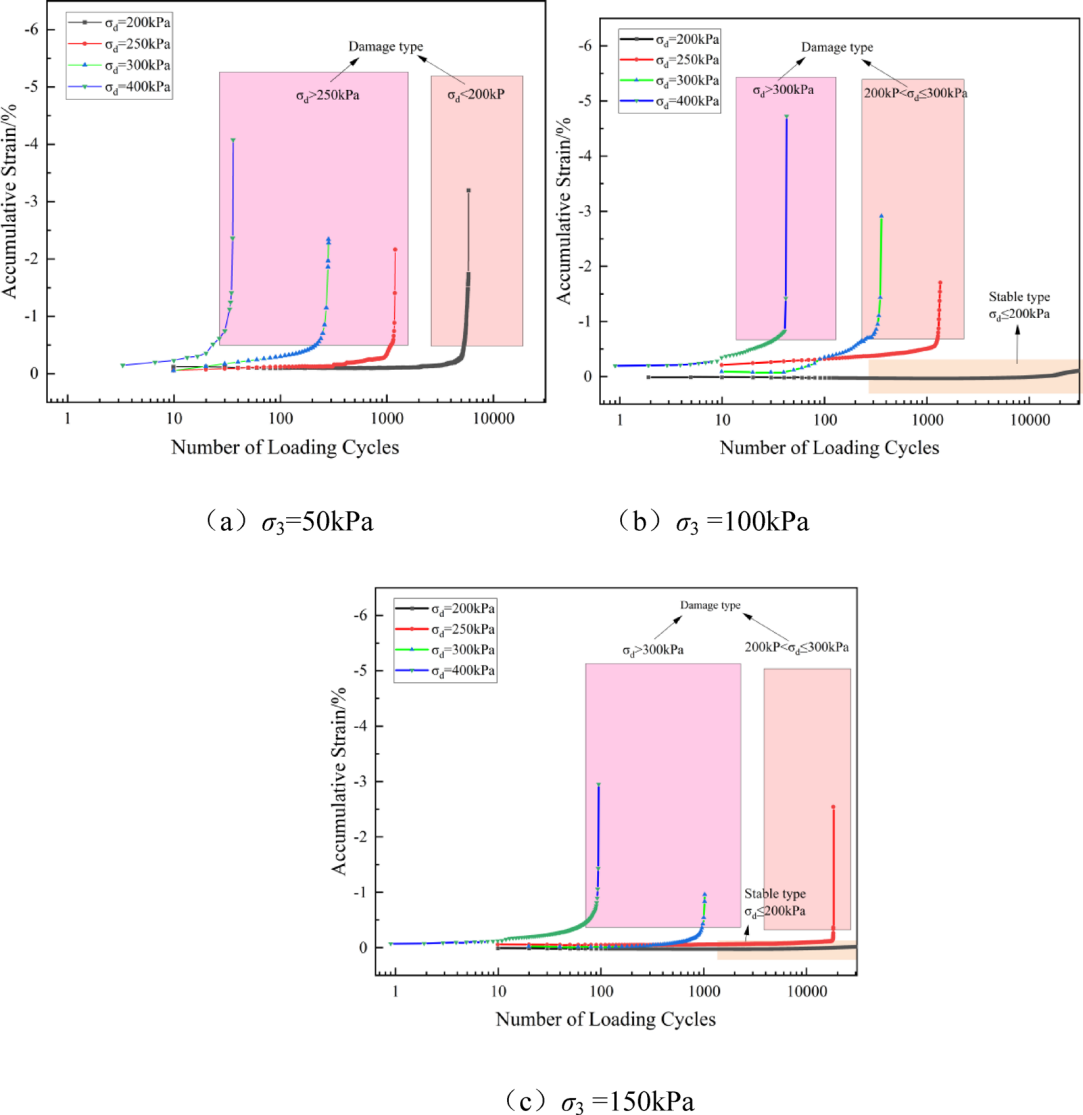
Relationship curve of cumulative strain-number of loading cycles vs. dynamic load amplitude at different confining pressures (aw = 5%).

Uneven settlement is a key factor affecting the service performance of the pavement and driving safety. Under long-term traffic loads, the cumulative plastic deformation of the subgrade filling material is one of the main causes of uneven settlement, which, in severe cases, can threaten driving safety^39^. Therefore, a thorough analysis of the cumulative deformation characteristics of solidified soil under cyclic loading is of significant engineering importance. Figures 6 and 7 show the relationship curves of the cumulative axial strain-load cycles of the solidified soil under different cement contents and confining pressures, as the dynamic load amplitude changes. In this study, the cumulative strain of the soil under compression is defined as negative.

Based on Figs. 6 and 7, the variations in the accumulated strain curve of solidified soil can be categorized into two types: stable and failure modes. The stable type is characterized by a slow and gradual increase in the accumulated dynamic strain, which eventually stabilizes as the number of loading cycles increases. In contrast, the failure type exhibits a nonlinear change in accumulated strain with an increase in the number of loading cycles, and after a certain loading threshold, the accumulated deformation increases rapidly, leading to the failure of the solidified soil specimen.

**Fig. 8:**
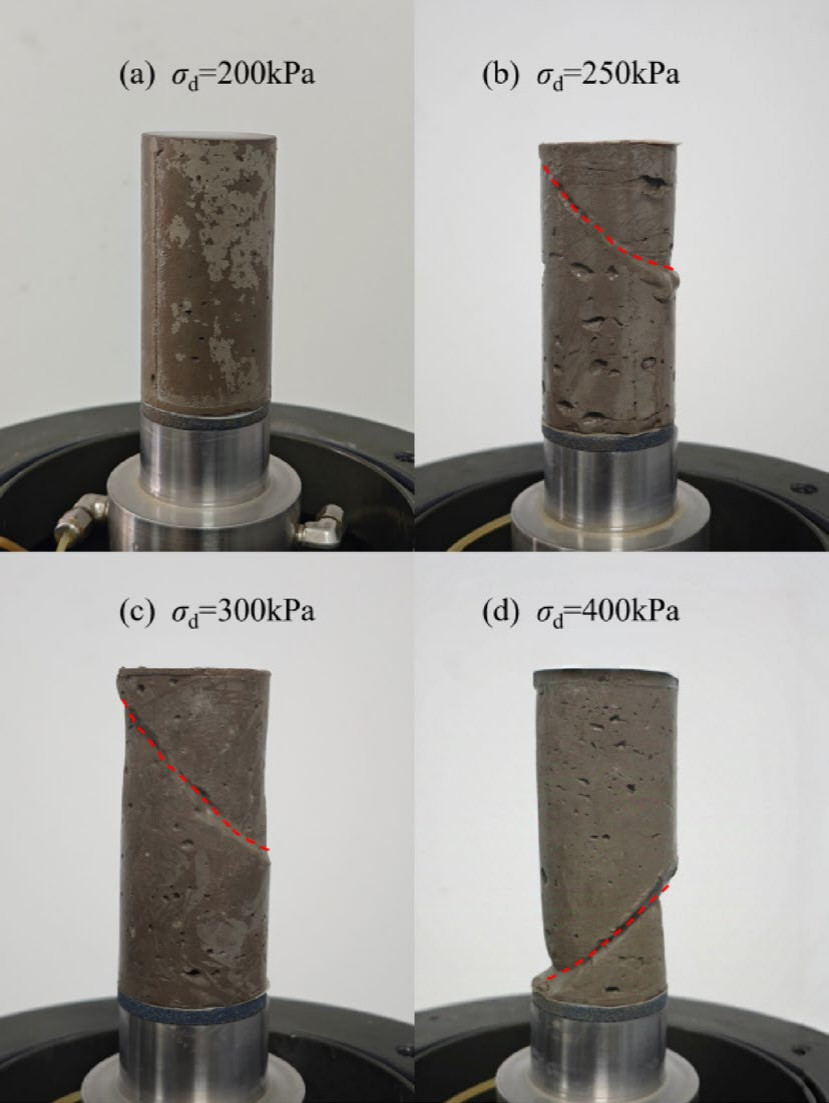
Sample failure states.

The failure types of solidified soil are shown in Fig. 8, where (a) represents the stable type, and (b), (c), and (d) represent the failure types. Based on the failure characteristics and failure mechanisms, the failure process of solidified soil can be divided into three stages:① Initial deformation stage: In the early cycles, strain increases rapidly, mainly due to particle reorganization and the compression of initial voids;② Stable deformation stage: The strain increase rate slows down and stabilizes, as the soil structure gradually adapts to cyclic loading, though internal micro-damage may accumulate slowly;③ Accelerated deformation stage: The strain increase rate sharply accelerates, and internal damage (such as micro-cracks) rapidly propagates and connects, leading to macroscopic failure of the specimen.

For the failure type, there exists a critical dynamic load *σ*_dcr_. When *σ*_d_ exceeds *σ*_dcr_, the strength of the specimen rapidly decreases^40^. When *a*_w_ = 4%, the critical dynamic load ranges from 100 to 200 kPa, and when aw exceeds 4%, the critical dynamic load ranges from 200 to 300 kPa. As the dynamic load amplitude increases, the accumulated strain of the specimen under the same cement content gradually increases for the same number of loading cycles, and the strain growth rate also accelerates^21^. The main reason for this is that under higher axial cyclic loading, the disturbance deformation of the specimen increases, and the internal pores and cracks continuously expand, leading to rapid development of accumulated plastic deformation.

**Fig. 9:**
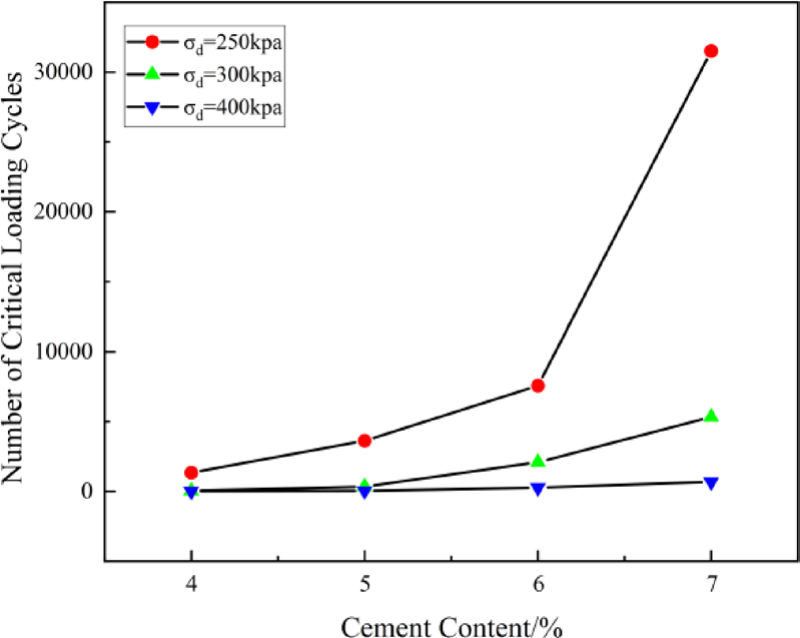
Relationship curve between cement content and critical loading cycles (*σ*₃ = 100 kPa).

As shown in Figs. 6 and 7, when the accumulated strain reaches 1%, the accumulated deformation of the specimen rapidly increases over a short period as the number of loading cycles increases. The number of loading cycles required for the specimen to reach an accumulated deformation of 1% is defined as the critical loading cycle. The cement content vs. critical loading cycle relationship curve is shown in Fig. 9 (with *σ*_3_ = 100 kPa as an example).

Figure 9 shows that as the cement content increases, the number of cycles to failure (*N*_fcr_) continues to rise, with a particularly significant increase under low dynamic loading (*σ*_d_ ≤ 250 kPa). The most substantial increase in *N*_fcr_ occurs when the cement content is between 6% and 7%. However, under higher dynamic loading (*σ*_d_ ≥ 400 kPa), the overall improvement in *N*_fcr_ is limited. Taking *σ*_3_ = 100 kPa as an example, when *σ*_d_ = 250 kPa, as *a*_w_ increases from 4 to 7%, the growth rates of *N*_fcr_ are 169.8%, 108.6%, and 316.3%, respectively. When *σ*_d_ = 300 kPa, the growth rates of *N*_fcr_ are 729.3%, 513.2%, and 155.4%, and when *σ*_d_ = 400 kPa, the growth rates are 300.2%, 575.1%, and 151.5%, respectively. The effect of increasing cement content on the improvement of critical loading cycles (*N*_fcr_) is initially enhanced and then diminishes. The rate of increase gradually slows due to the limitation imposed by dynamic loading. This is because higher dynamic loading accelerates the formation and propagation of microcracks within the sample, weakening the fatigue resistance of the solidified soil. The failure of the solidified soil becomes more dependent on the magnitude of the external load, and the role of cement content in delaying deformation becomes relatively reduced. The variation of critical dynamic stress (*σ*_dcr_) and critical loading cycles (*N*_fcr_) with cement content and stress levels revealed in this study provides a basis for assessing the long-term service performance of solidified soil and guiding related engineering design.

The variation of critical dynamic stress (*σ*_dcr_) and critical loading cycles (*N*_fcr_) with cement content and stress levels, as revealed in this study, provides valuable insights for assessing the long-term service performance of solidified soil and guiding related engineering design.

### Initial dynamic elastic modulus

The ratio of the stress difference to the strain difference at the endpoints of a hysteresis loop within a vibration cycle is defined as the dynamic modulus of elasticity (*E*_d_). It reflects the soil’s ability to resist deformation and its stiffness^41^. Typically, the slope of the hysteresis loop under a cyclic load represents the dynamic modulus of elasticity (*E*_d_), and its formula is as follows:

7 $${E_{\text{d}}}=\frac{{{\sigma _d}}}{{{\varepsilon _d}}}=\frac{{{\sigma _{_{{\hbox{max} }}}} - {\sigma _{\hbox{min} }}}}{{{\varepsilon _{\hbox{max} }} - {\varepsilon _{\hbox{min} }}}}$$

In the equation:

*σ*_max_ and *σ*_min_ represent the maximum and minimum axial dynamic loads, respectively, under the same cyclic loading, in units of kPa.

*ε*_max_ and *ε*_min_ represent the maximum and minimum axial dynamic strains, respectively, under the same cyclic loading, in units of %.

**Fig. 10:**
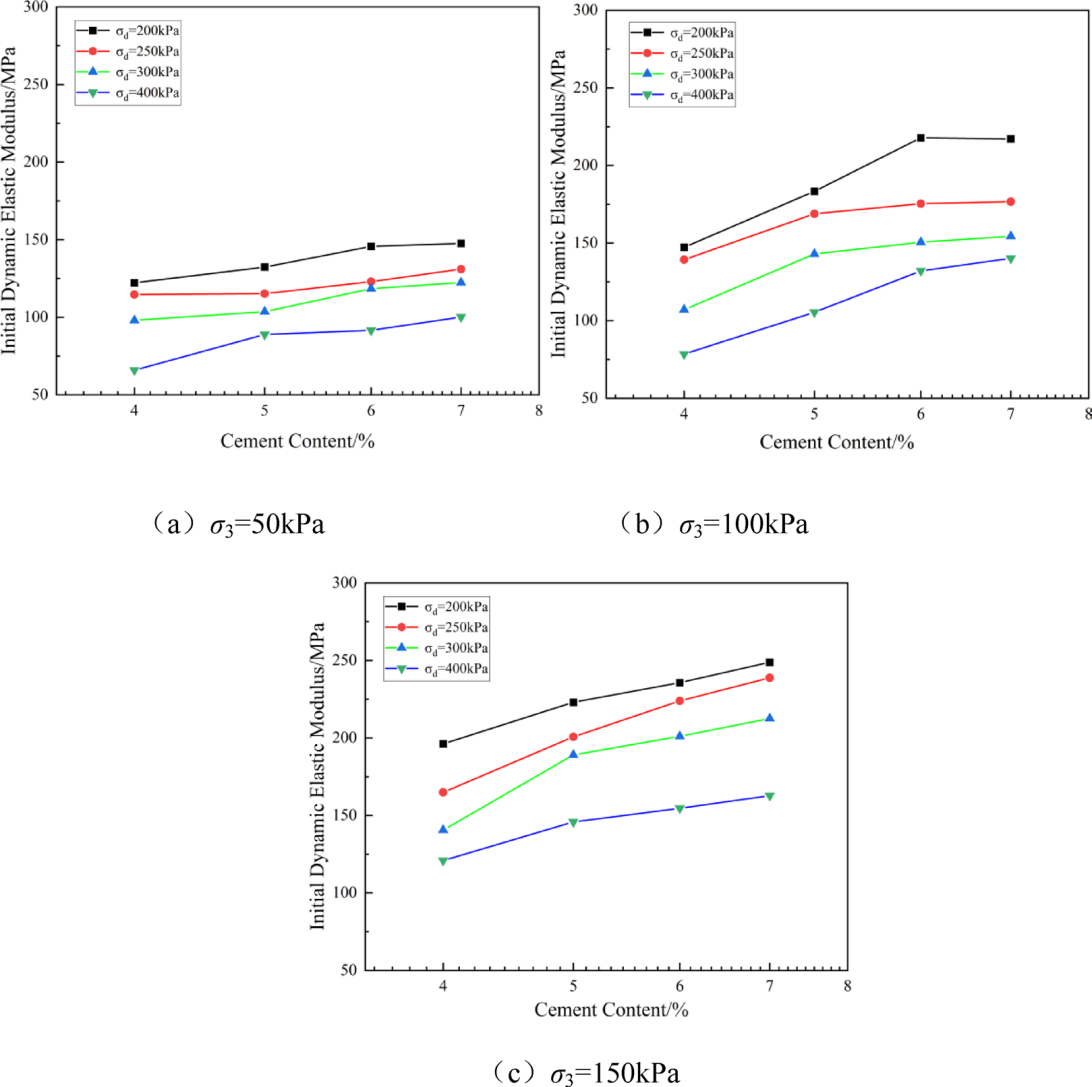
Relationship between initial dynamic elastic modulus of solidified soil and cement content.

**Fig. 11:**
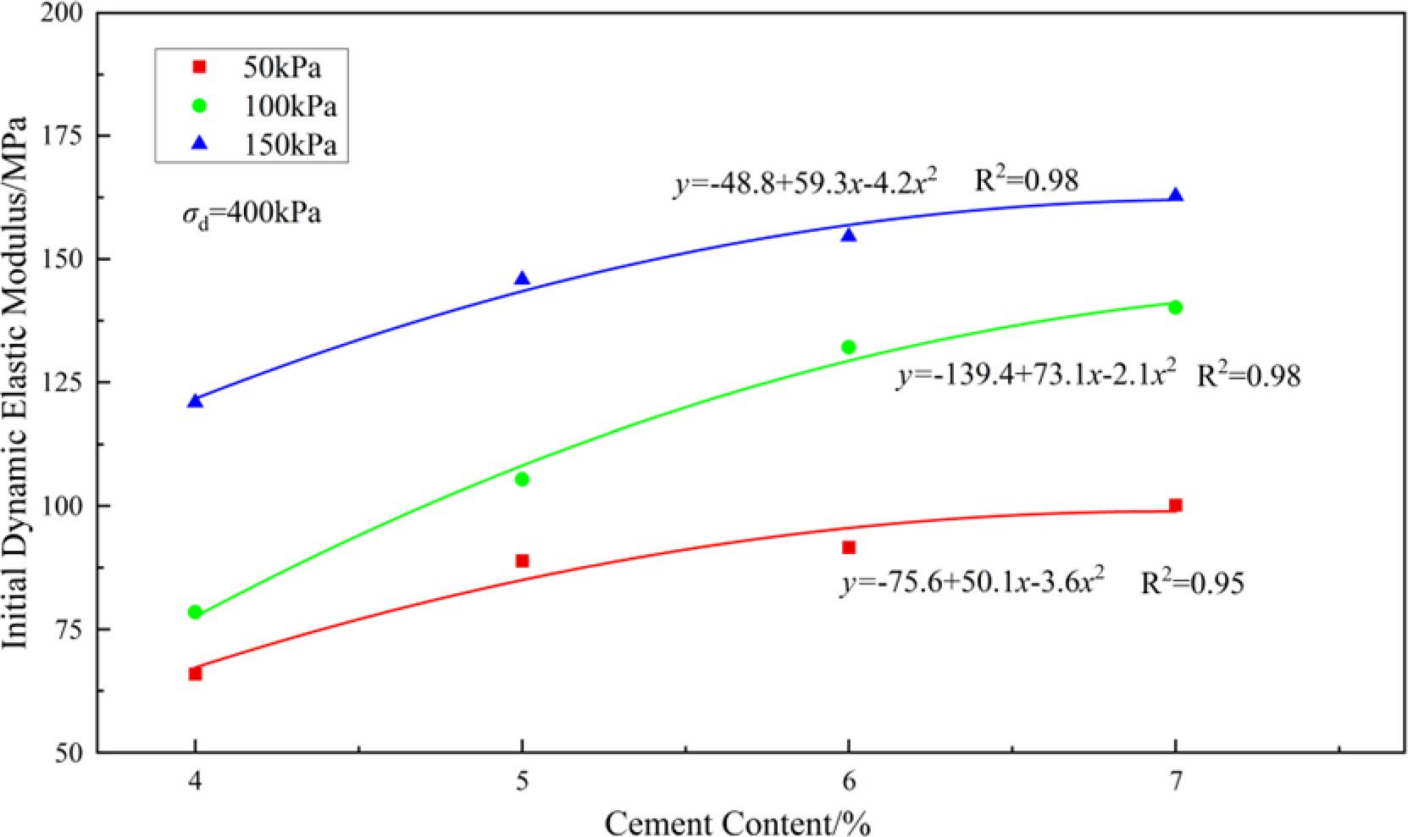
Fitting curve of initial dynamic elastic modulus of solidified soil vs. cement content.

Figure 10 shows the relationship between the initial dynamic modulus of solidified soil and cement content, obtained from the experiments. Figure 11 presents the fitted curve of the initial dynamic modulus of solidified soil versus cement content.

As shown in Figs. 10 and 11, the initial dynamic modulus increases with higher cement content, particularly in the lower cement content range (4–6%), where the modulus grows rapidly (average growth rate of 12.9–39.3%). However, when the cement content exceeds 6%, the increase becomes more gradual, with growth rates between 0.3% and 5% from 6 to 7%. Cement content is a critical factor influencing the fatigue life of cement-stabilized soil. However, the diminishing growth rate of the initial dynamic modulus with increasing cement content suggests the existence of an optimal cement content for enhancing the modulus. This finding aligns with previous conclusions proposed by scholars such as Zhang Minxia^30^ and Janbaz^42^, who also reported the presence of an optimal cement content. Confining pressure also significantly affects the variation of the initial dynamic modulus. Under low confining pressure (50 kPa), the modulus varies by 35–55% across different deviatoric stresses. However, as the confining pressure increases, the variation range narrows to 25–37%.At a constant cement content, the weakening effect of dynamic load on the modulus is more pronounced under low confining pressure (50 kPa), while this effect is reduced under high confining pressure (150 kPa). Increased confining pressure enhances the overall initial dynamic modulus, and as confining pressure rises, the influence of both cement content and dynamic loading becomes less significant.

A power function fitting was performed between the unconfined compressive strength (*q*_u_) and the initial dynamic modulus (*E*_d_) of solidified soil under different σd values at a confining pressure of *σ*_3_ = 100 kPa. The fitting equation is as follows:

8 $$\ln \left( {E_{d} } \right) = \ln \left( a \right) + b \cdot \ln \left( {q_{u} } \right)$$

Where: *a* and *b* are constants. The detailed values of the fitting parameters *a* and *b* are shown in Table 4 below.

**Table 4:** Fitting parameters of dynamic elastic modulus for solidified soil.

Dynamic Load /kPa	ln(a)	Fitting Parameter b	*R* ^2^
200	5.56	0.60	0.89
250	5.29	0.35	0.83
300	5.20	0.54	0.85
400	5.17	0.88	0.94

Regarding the mechanism by which increased cement content affects the initial dynamic modulus, the analysis suggests that at low cement contents, the amount of hydration-induced cementitious products is limited, resulting in insufficient bonding between clay particles. Therefore, increasing the cement content promotes the formation of more hydration products, enhancing the initial dynamic modulus of the specimen. This observation is consistent with the findings of Sun Haichao et al.^9^.

## Conclusion

This study experimentally investigated the dynamic behavior of typical silty clay sludge from Ningbo after stabilization with low cement content. The effects of varying cement content, confining pressure, and dynamic loading on the dynamic strength, accumulated plastic deformation, and initial dynamic modulus of the stabilized soil were systematically analyzed. The main conclusions are as follows:

1. Unconfined compressive strength tests indicated that the threshold cement content (*a*_wc_) for 28-day cured sludge-stabilized soil is 7.0%. Within the low cement content range (*a*_w_ < *a*_wc_), strength development was relatively slow and nonlinear. In contrast, beyond this threshold (*a*_w_ > *a*_wc_), strength increased significantly and tended toward linearity, with the average strength growth rate being 1.88 times higher than that in the low-content range.
2. The dynamic strength of the stabilized sludge soil exhibits a strong correlation with the number of loading cycles. A unified predictive model for dynamic strength was developed in this study, which can assist engineers in estimating the dynamic strength behavior of stabilized sludge with varying cement contents. This, in turn, enables the determination of the critical failure cycles under different dynamic loads, providing a valuable reference for fatigue-resistant design of subgrade structures and for preliminary selection of cement content.
3. The accumulated plastic deformation behavior of the stabilized soil indicates a strong correlation between cement content (*a*_w_) and the critical number of failure cycles (*N*_fcr_). When *a*_w_ increased from 4 to 6%, *N*_fcr_ rose significantly—by up to 316.3% under low dynamic loads and 575.1% under high dynamic loads. However, beyond 6% cement content, the growth rate of *N*_fcr_ declined sharply, with increases of only 155.4% under low dynamic loads and 151.5% under high loads. This suggests that once the cement content surpasses a certain threshold, its effectiveness in delaying deformation diminishes, and the fatigue resistance enhancement gradually plateaus. These findings highlight the importance of considering the marginal benefit of cement dosage in engineering design.
4. The initial dynamic modulus of the stabilized soil increased significantly with higher cement content, particularly within the 4–6% range. Beyond 6%, the rate of increase gradually diminished. As the dynamic modulus is a key indicator of subgrade stiffness, the findings of this study provide important insights into how cement content influences the early-stage stiffness of stabilized soils.
5. This study provides essential design parameters and performance prediction methods for the application of low-cement-content sludge-stabilized soil in subgrade engineering. Taking into account both the dynamic performance under long-term cyclic loading and the cost-effectiveness of cement usage, a cement content of 6–7% is recommended. Within this range, the stabilized soil demonstrates favorable performance in terms of dynamic strength, resistance to accumulated deformation, and initial dynamic modulus. This level of performance meets the stability and durability requirements for most subgrade projects while avoiding excessive economic costs and potential environmental impacts associated with higher cement usage.
6. Future research could further explore the effects of different loading frequencies and waveforms on the dynamic properties of low-cement-content sludge-stabilized soil to more accurately simulate the diversity of real-world traffic loads. Additionally, the potential of partially replacing cement with industrial by-products (such as fly ash or ground granulated blast furnace slag) or novel eco-friendly binders should be investigated. Studying the dynamic behavior of such stabilized soils, along with long-term environmental assessments and in-situ engineering validations, would contribute to expanding the pathways for resourceful sludge utilization and promoting its large-scale implementation in engineering projects.

## Data Availability

The data that support the findings of this study are available from the corresponding author upon reasonable request.
